# Monoamine Oxidase-B Inhibitor Reduction in Pro-Inflammatory Cytokines Mediated by Inhibition of cAMP-PKA/EPAC Signaling

**DOI:** 10.3389/fphar.2021.741460

**Published:** 2021-11-17

**Authors:** Edward E. Putnins, Verena Goebeler, Mahyar Ostadkarampour

**Affiliations:** ^1^ Department of Oral Biological and Medical Sciences, Faculty of Dentistry, The University of British Columbia, Vancouver, BC, Canada; ^2^ Department of Pediatrics, Faculty of Medicine, The University of British Columbia, Vancouver, BC, Canada

**Keywords:** monoamine oxidases, monoamine oxidase inhibitors, lipopolysaccharide, inflammation, cytokines, cAMP, protein kinase A (PKA), exchange protein activated by cAMP (EPAC)

## Abstract

Mucosal epithelial cell integrity is an important component of innate immunity and it protects the host from an environment rich in microorganisms. Virulence factors from Gram-negative bacteria [e.g. lipopolysaccharide (LPS)] induce significant pro-inflammatory cytokine expression. Monoamine oxidase (MAO) inhibitors reduce cytokine expression in a variety of inflammatory models and may therefore have therapeutic potential for a number of inflammatory diseases. We tested the anti-inflammatory therapeutic potential of a recently developed reversible MAO-B inhibitor (RG0216) with reduced transport across the blood–brain barrier. In an epithelial cell culture model, RG0216 significantly decreased LPS-induced interleukin (IL)-6 and IL-1β gene and protein expression and was as effective as equimolar concentrations of deprenyl (an existing irreversible MAO-B inhibitor). Hydrogen peroxide and modulating dopamine receptor signaling had no effect on cytokine expression. We showed that LPS-induced expression of IL-6 and IL-1β was cAMP dependent, that IL-6 and IL-1β expression were induced by direct cAMP activation (forskolin) and that RG0216 and deprenyl effectively reduced cAMP-mediated cytokine expression. Targeted protein kinase A (PKA) and Exchange Protein Activated by cAMP (EPAC) activation regulated IL-6 and IL-1β expression, albeit in different ways, but both cytokines were effectively decreased with RG0216. RG0216 reduction of LPS-induced cytokine expression occurred by acting downstream of the cAMP-PKA/EPAC signaling cascade. This represents a novel mechanism by which MAO-B selective inhibitors regulate LPS-induced IL-6 and IL-1β expression.

## Introduction

The mucosal epithelium provides a functional barrier in areas such as the oral cavity, gastrointestinal track, lungs and reproductive tracts. Barrier integrity is a component of innate immunity and its disruption initiates a cascade of immune defense responses (Geremia et al., 2014; Kugelberg, 2014; Barrios De Tomasi et al., 2019; Boonpiyathad et al., 2019; Groeger and Meyle, 2019). Innate immune responses are triggered by unique pathogen-associated molecular patterns (PAMPs) and damage-associated molecular patterns (DAMPs) molecules (Heijink et al., 2015; Groeger and Meyle, 2019). Induction of P/DAMP signaling drives epithelial cells to express pro-inflammatory cytokines and chemokines such as interleukin-1*β* (IL-1β), tumor necrosis factor-*α* (TNF-α), interleukin-6 (IL-6) and interleukin-8 (IL-8) (Borish and Steinke, 2003; Kugelberg, 2014; Groeger and Meyle, 2019; Plemmenos et al., 2021). Significant evidence supports that reduction of monoamine oxidase enzyme function by MAO inhibitors decreases innate immune-based cytokine signaling and positively improves disease progression (Ostadkarampour and Putnins, 2021).

Monoamine oxidases are mammalian flavoenzymes that are associated with the mitochondrial membrane and catalyze deamination of biogenic and dietary amines, monoamine hormones, neurotransmitters such as serotonin, dopamine, (nor)epinephrine and trace amines such as tyramine, tryptamine and 2-phenylethylamine (Youdim et al., 2006; Bortolato et al., 2008; Edmondson and Binda, 2018; Tipton, 2018). Monoamine oxidases exist in two isoforms (MAO-A and MAO-B), each with different substrate specificities, inhibitor affinity, localization and expression (Tipton, 2018). MAO-A has high affinity and catalyzes the oxidation of serotonin; MAO-B oxidizes 2-phenylethylamine and benzylamine. Dopamine, (nor)epinephrine, tryptamine and tyramine are oxidized by both (Youdim et al., 2006).

Clinically, reversible and irreversible MAO-A, MAO-B and MAO-A/B inhibitors are prescribed for the management of depression, anxiety disorders, Parkinson’s and Alzheimer’s diseases (Youdim et al., 2006; Bortolato et al., 2008; Tipton, 2018). A number of studies have also demonstrated that MAO inhibitors decrease gene and protein expression of a number of pro-inflammatory cytokines and chemokines. This reduction in drivers of inflammation may have therapeutic potential (Ostadkarampour and Putnins, 2021).

MAO inhibitors are MAO-A, MAO-B or MAO-A/B selective and bind in a reversible or irreversible manner. They reduce metabolic end products such as hydrogen peroxide, aldehydes and ammonium but also increase levels of catecholamines such as dopamine (Eisenhofer et al., 2004; Ostadkarampour and Putnins, 2021). Dopamine modulates inflammatory responses in non-neuronal cells such as lymphocytes and epithelial cells as well (Bergquist et al., 1994; Goldstein et al., 1995; Adir et al., 2004; Qiu et al., 2005; Matt and Gaskill, 2020). MAO-B inhibitors (rasagiline and deprenyl) and an MAO-A inhibitor (clorgyline) increased dopamine and norepinephrine in rat pheochromocytoma cells (Goldstein et al., 2016). Cell response to dopamine is dependent on receptor type, relative expression and state of cell activation (Matt and Gaskill, 2020). Dopamine signals are mediated through a family of receptors that are grouped into two subclasses: D1-like (DR1 and DR5) and D2-like (DR2, DR3 and DR4) (Beaulieu and Gainetdinov, 2011; Beaulieu et al., 2015). D1-like receptors are coupled to G_αs_ and stimulate production of cAMP. Conversely, D2-like receptors inhibit cAMP production (Beaulieu and Gainetdinov, 2011; Beaulieu et al., 2015).

Cyclic AMP is a ubiquitous mediator of inflammation and modulates the immune response (Raker et al., 2016; Tavares et al., 2020). It serves as a second messenger to mediate physiological cell function. Cyclic AMP in turn can induce activation of protein kinase A (PKA) as well as Exchange Protein Activated by cAMP (EPAC1 and EPAC2) (Beaulieu et al., 2015). PKA and EPAC can both activate/inhibit the Ras/Rap-Raf-MEK-ERK pathway with their subsequent independent, synergic or antagonistic interactions, which regulates the varied cellular responses that are generated (de Rooij et al., 1998; Stork and Schmitt, 2002; Bos, 2003, Bos, 2006; Cheng et al., 2008; Miningou and Blackwell, 2020). LPS-induced TNF-α expression in macrophages was only inhibited by PKA and not EPAC activation (Bryn et al., 2006). In microglial cells, PKA activation reduced TNF-α but increased IL-1β expression, and IL-6 expression was reduced with a PKA selective inhibitor in murine mesangial cells (Grassl et al., 1999; Woo et al., 2003). In contrast, only EPAC activation decreased LPS-induced keratinocyte-derived chemokines (KC, CXCL1) in murine alveolar epithelial cells (Wang et al., 2018b). Altogether, the regulation of cytokine expression through cAMP-PKA/EPAC signaling is complex, highly variable and cell specific.

The repurposing of existing, and development of novel, MAO inhibitors have been proposed for the treatment of cancer, cardiovascular disease, muscular dystrophy and treatment of chronic inflammatory diseases (Deshwal et al., 2017; Carradori et al., 2018; Shih, 2018; Vitiello et al., 2018). The potential that MAO inhibitors may be effective in non-central nervous system (CNS) chronic inflammatory environments has led to the early development of MAO-B inhibitors with reduced blood–brain permeability (Gealageas et al., 2018). One lead (compound 10j, recoded as RG0216) was a reversible MAO-B inhibitor with submicromolar activity against MAO-B and no effect on MAO-A (Supplemental Figure S1). We examined the *in vitro* anti-inflammatory potential of RG0216 using a cervical epithelial cell line (SiHa) that only expresses MAO-B, and LPS was used as the innate immune driver of inflammation. In addition, we examined the signaling mechanisms by which LPS induced IL-6 and IL-1β expression and its inhibition by RG0216 and deprenyl.

## Materials and Methods

### Cell Culture Reagents and Chemicals

A human cervical mucosal epithelial cell line (SiHa) (ATCC HTB-35) was cultured in *α*MEM medium (Gibco) containing 10% fetal bovine serum (FBS) (Gibco), 50 units/mL penicillin and 50 μg/ml streptomycin (Gibco) and maintained at 37°C with 5% CO_2_. Cultures, when 75% confluent, were starved overnight in serum-free medium. Cells were stimulated with *E. coli* LPS (O55:B5) (Sigma) at 25 μg/ml in serum-free media. To examine the effect of MAO inhibitors on cytokine expression ± LPS-treated cultures were preincubated for 1 h with the MAO-B inhibitors RG0216 or deprenyl hydrochloride (M003, Sigma-Aldrich). The effects of LPS, RG0216 and hydrogen peroxide on cell viability was assayed using the CellTiter 96^®^ AQ_ueous_ One Solution Cell Proliferation Assay kit (Promega). Briefly, 1.2 × 10^4^ cells/well were plated in 96-well plates and cultured to 80% confluence. Treated cells were cultured for 4 h and the CellTiter 96^®^ AQ_ueous_ One Solution Reagent added directly to culture wells and incubated at 37°C for 1 h in a humidified, 5% CO_2_ atmosphere. The absorbance was read using a FLUOstar Omega (BMG LABTECH, Germany) reader at 490 nm.

The regulation of cytokine expression by increased hydrogen peroxide was examined using 30% hydrogen peroxide (VWR Chemical BDH^®^) at a final concentration of 10 and 100 µM. The potential role of dopamine receptor signaling in the regulation of cytokine expression was examined using dopamine receptor class-specific agonists/antagonists. Specifically, the D1-like agonist was dihydrexidine hydrochloride (1 μM, Tocris) and the antagonist was SCH 23390 hydrochloride (1 μM, Tocris). The D2-like agonist was sumanirole maleate (1 μM, Tocris) and the antagonist was L-741,626 (1 μM, Tocris). The role of cAMP-PKA/EPAC signaling in the regulation of cytokine expression was examined using pathway selective activators and inhibitors (Tocris). Cyclic AMP signaling was examined using a cAMP activator (10 μM, forskolin) (Kim et al., 2005) or cAMP inhibitor (100 μM, SQ 22536) (Weber et al., 2018). PKA/EPAC signaling was examined using either the PKA activator (100 μM, 6-Bnz-cAMP sodium salt) (Lo et al., 2016) or the EPAC activator (10 μM, 8-pCPT-2-O-Me-cAMP-AM) (Chepurny et al., 2009).

### MAO-B Enzyme Activity Assay

MAO-B enzyme activity in SiHa cells was assayed using a commercial Monoamine Oxidase B detection kit (Cell Technology). Protein cell lysate from 20 million cells was prepared using RIPA lysis buffer containing a protease inhibitor cocktail (ChemCruz). Serially diluted protein lysate samples (1–64 μg/ml, *n* = 2) were added to the reaction buffer with ± RG0216 (20 and 100 µM) 30 min prior to adding an MAO-B selective substrate (2.5 mM final benzylamine) and incubated at room temperature for an additional 30 min. MAO-B oxidation of benzylamine generates as an end product hydrogen peroxide, which oxidizes the detection reagent in 1:1 stoichiometry. A fluorescent product is produced by horseradish peroxidase (HRP) and detected using a FLUOstar Omega (BMG LABTECH) reader with excitation and emission wavelengths of 570 and 590 nm, respectively. MAO-B enzyme activity is presented as Relative Fluorescence Units (RFU).

### Cytokine Protein Expression

Protein expression of IL-1β, IL-6, TNF-α and IL-8 were quantified by electrochemiluminescence using Meso Scale Discovery V-PLEX on a Meso Quickplex SQ 120 (Model 1300) reader (Meso Scale Discovery, Rockville, Maryland, United States). Briefly, 1.2 × 10^4^ cells were cultured in 96-well plates (*n* = 4) and serum-starved overnight. Cultures were treated with ± MAO inhibitors (RG0216 or deprenyl) added 1 h prior to the addition of LPS. cAMP activators/inhibitors or PKA and EPAC activators were added in conjunction with RG0216. Conditioned media was collected at 12 h post LPS addition and cytokines were assayed following the manufacturer’s protocol.

### Gene Expression by RT-qPCR

To cell cultures (2 × 10^5^ cells), DR agonists/antagonists or cAMP activators/inhibitors or PKA and EPAC activators were added in conjunction with RG0216 or deprenyl and all were added 1 h prior to the addition of LPS. Cells were harvested at 5 h (4 h after LPS addition). RNA was extracted using the PureLink RNA Mini Kit (Invitrogen), RNA was normalized to 2 µg by NanoDrop One^c^ (Thermo Scientific) and reverse transcribed to cDNA using SuperScript VILO Master Mix (Invitrogen). cDNA was added to MicroAmp^®^ EnduraPlate Optical 96-well Fast Clear reaction plates (Applied Biosystems) and real-time PCR completed using TaqMan™ Fast Advanced Master Mix (Applied Biosystems™). To analyze gene expression, the following PCR primers and probes were used: MAO-A (Hs00165140_m1), MAO-B (Hs01106246_m1), IL-6 (Hs00174131_m1), IL-1*β* (Hs01555413_m1), DR1 (Hs00265245_s1), DR2 (Hs00241436_m1), DR3 (Hs00364455_m1), DR4 (Hs00609526_m1), DR5 (Hs00361234_s1), tyrosine hydroxylase (Hs00165941_m1), dopamine decarboxylase (Hs01105048_m1) and GAPDH (Hs02786624_g1) used as an endogenous control (Thermo Fisher). Quantitative PCR (qPCR) was performed on the QuantStudio three Thermocycler (Applied Biosystems). RNA expression was calculated (2^−ΔCt^) and relative fold change in gene expression in relation to unstimulated controls was calculated using the comparative Ct method (2^−ΔΔCt^).

### Western Blot

SiHa cell line expression of MAO-A and -B protein were examined using Western blot. Cells in a T25 flask were cultured to 80% confluency and lysed using RIPA lysis buffer containing a protease inhibitor cocktail, PMSF (phenylmethylsulfonyl fluoride) and sodium orthovanadate (ChemCruz). Protein was quantified using the BCA™ Protein Assay Kit (Thermo Fisher Scientific) and 50 µg of total protein loaded in each well of a 10% polyacrylamide gel. Human recombinant MAO-A (Sigma M7316) and human recombinant MAO-B (Sigma M7441) were included as positive controls. Proteins were wet-transferred onto PVDF (polyvinylidene difluoride) membrane, blocked using Immobilon^®^ Block-FL (Fluorescent Blocker) (Millipore) for 1 h and incubated with primary antibodies overnight at 4°C, then rinsed and incubated with secondary antibodies for 1 h at room temperature. The following primary and secondary antibodies were used: rabbit monoclonal anti-MAO-A (Abcam, ab126751), rabbit polyclonal anti-MAO-B (Picoband™, Boster Bio, PB9665), mouse monoclonal anti-Actin [ACTN05 (C4)] (Abcam ab3280), donkey anti-rabbit IRDye 800CW (Licor, 926–32,213), and donkey anti-mouse IRDye 680RD (Licor, 906–68,072). Protein bands were visualized using the LI-COR Odyssey^®^ scanner and relative protein expression of MAO-A and -B were examined. Actin served as an endogenous loading control.

### Statistical Analysis

Multiple comparisons between groups were examined using one-way analysis of variance (ANOVA) followed with Tukey’s post-test analysis. A *p*-value of less than 0.05 was considered statistically significant. All statistical analyses were performed with GraphPad PRISM 9.0.2 (GraphPad Software Inc.).

## Results

### MAO-B Expression in SiHa Cells Is Not Induced by LPS

**FIGURE 1:**
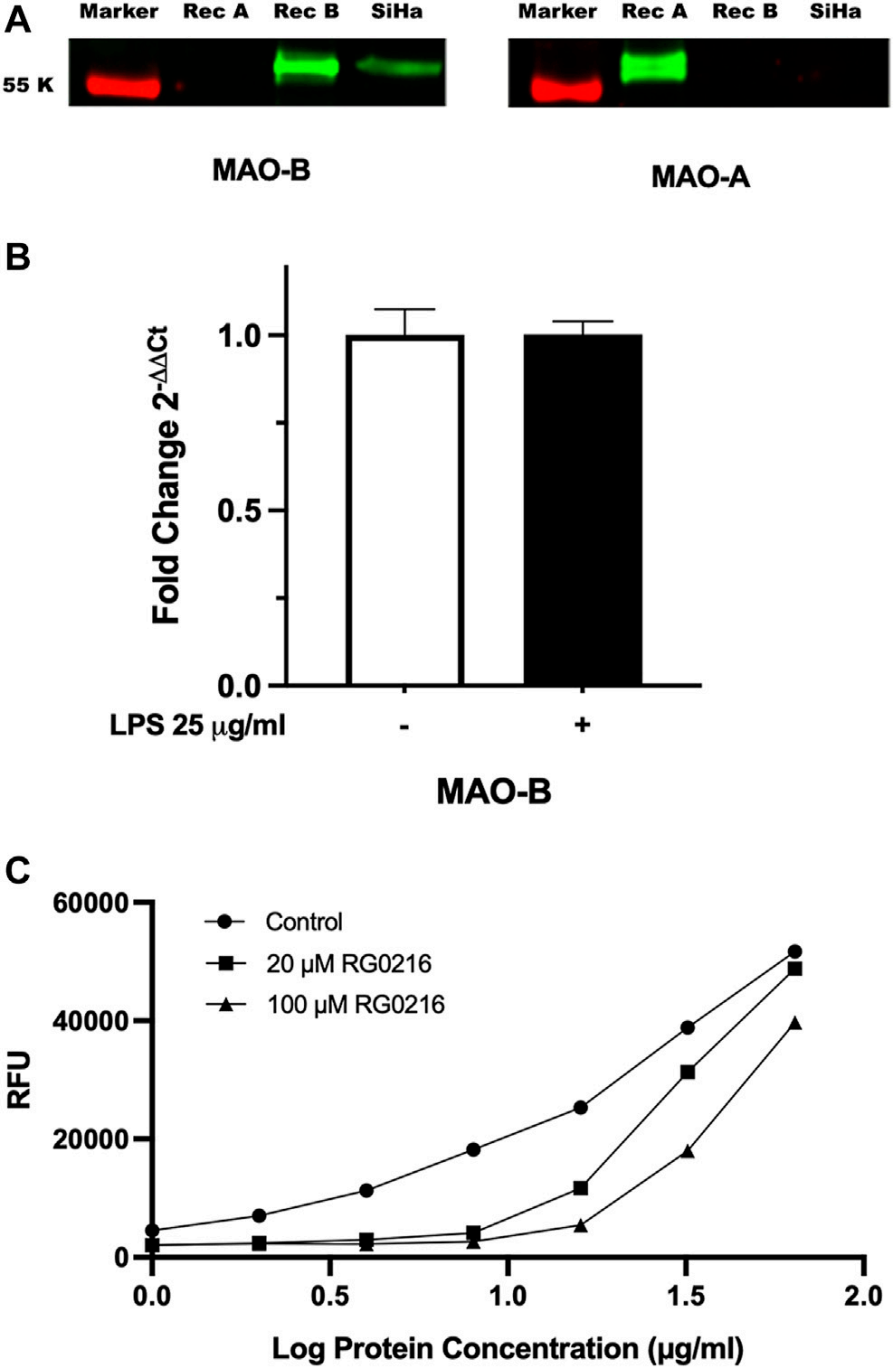
MAO-B protein is only expressed in SiHa cells. **(A)** MAO-A and MAO-B protein expression was examined in cell lysates using SDS-PAGE and Western blotting. Recombinant MAO-A (Rec A) and MAO-B (Rec B) proteins included as positive control. Left blot stained with anti-MAO-B primary antibody and right blot stained with anti-MAO-A primary antibody. Only MAO-B protein was expressed. **(B)** LPS stimulation had no effect on MAO-B relative gene expression. Data presented as mean ± SD (*n* = 3). **(C)** RG0216 inhibits MAO-B functional activity in SiHa cell lysates. MAO-B enzyme activity represented as Relative Fluoresence Units (RFU). Data presented as mean (*n* = 2).

Only MAO-B protein was expressed in SiHa cell lysates (Figure 1A). In control cultures, MAO-B gene expression was 260-fold higher than MAO-A (MAO-B/MAO-A 2^-∆Ct^ ratio) (data not shown) and LPS stimulation did not alter MAO-B gene expression (Figure 1B). We tested RG0216 inhibition of MAO-B enzymatic activity in SiHa cell lysates using an MAO-B selective substrate (benzylamine). RG0216 (20 and 100 µM) fully inhibited MAO-B activity in cell lysate preparations up to 8 μg/ml (log_10_ 0.9). At higher protein lysate concentrations of up to 64 μg/ml (log_10_ 1.8), RG0216 paritally inhibited MAO-B activity; however, at all concentrations tested, 100 µM was more effective (Figure 1C). Collectively, SiHA cells only express MAO-B protein and RG0216 inhibits MAO-B enzyme activity on SiHa cell lysates.

### MAO-B Inhibitor Reduced LPS-Induced Pro-Inflammatory Cytokine Expression

**FIGURE 2:**
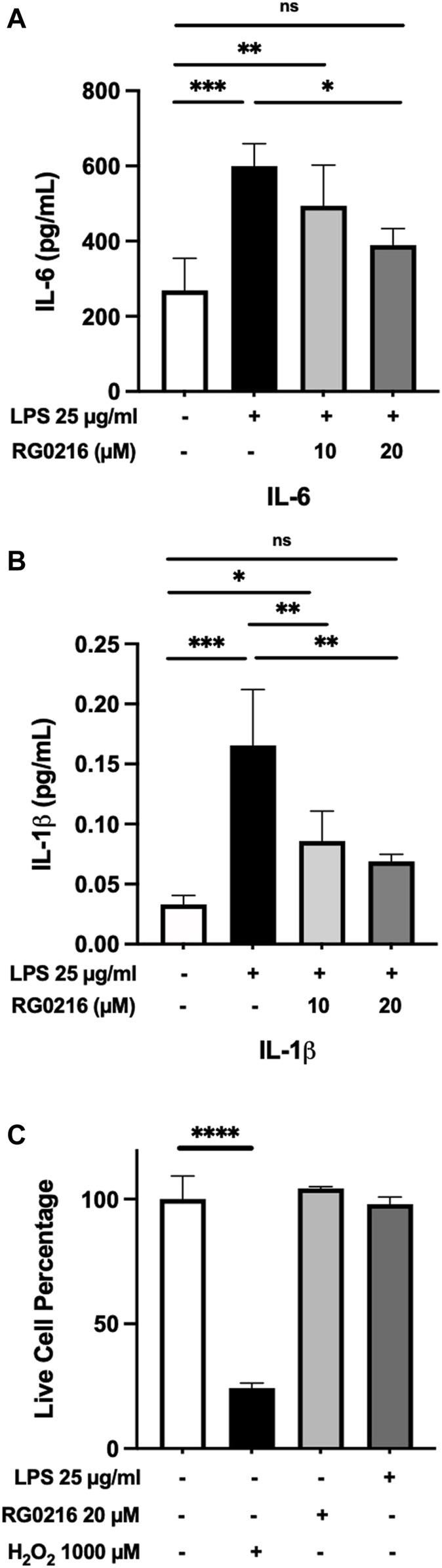
LPS-induced protein expression of IL-6 and IL-1β in SiHa cells was reduced in a concentration-dependent manner by RG0216. Both IL-6 **(A)** and IL-1β **(B)** protein expression were significantly increased by LPS and both maximally reduced with 20 μM of RG0216. **(C)** RG0216 and LPS did not affect cell viability but a high concentration of hydrogen peroxide (1,000 µM) reduced cell viability (control). Data presented as mean ± SD (*n* = 4, gene analysis; *n* = 3, viability assay). ns, not significant, *p < 0.05, **p < 0.01, ***p < 0.001 and ****p < 0.0001.

**FIGURE 3:**
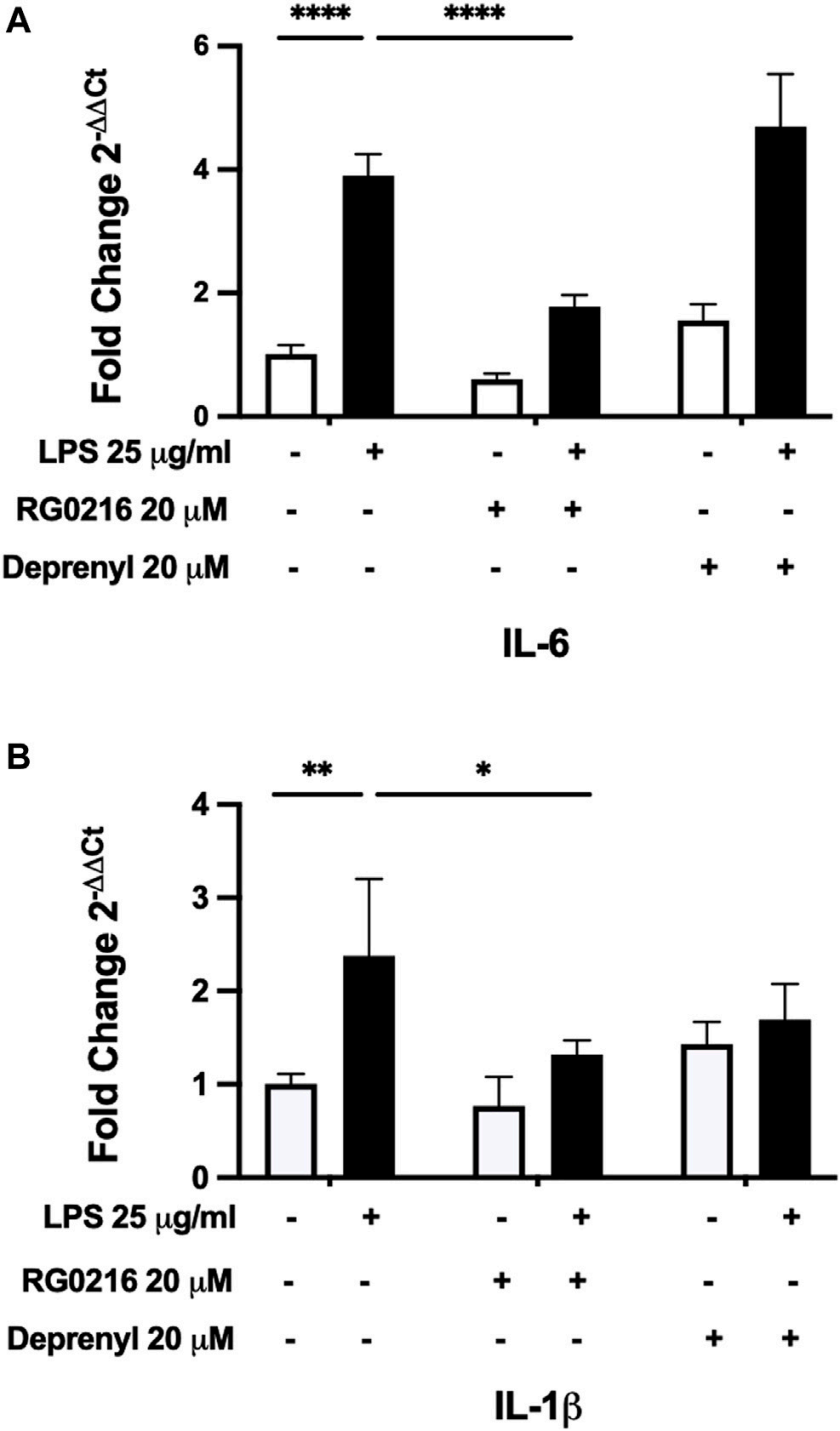
RG0216 and deprenyl regulation of LPS-induced IL-6 and IL-1β gene expression. Neither IL-6 **(A)** nor IL-1β **(B)** gene expression in control cultures (white bar) were significantly altered by RG0216 or deprenyl. LPS significantly induced gene expression of both pro-inflammatory cytokines (black bars); however, only RG0216 significantly reduced their gene expression at 4 h. The analysis was done using qRT-PCR. Data presented as mean ± SD (*n* = 4). *p < 0.05, **p < 0.01 and ****p < 0.0001.

LPS induced the secretion of IL-8, TNF-α, IL-1β and IL-6. LPS-induced secretion of IL-8 and TNF-α were not significantly reduced by RG0216 (data not shown and excluded from further study). LPS-induced IL-6 and IL-1β protein secretion were both significantly reduced by RG0216 treatment in a concentration-dependent manner (Figures 2A,B). Relative change in cytokine expression was not reflective of any RG0216- and LPS-mediated effect on cell numbers (Figure 2C). RG0216 and deprenyl regulation of IL-6 and IL-1β gene expression was subsequently examined. RG0216 and deprenyl had no significant effect on baseline IL-6 and IL-1β gene expression (Figures 3A,B). LPS-induced IL-6 and IL-1β expression were significantly reduced with RG0216 (20 µM). An equimolar concentration of deprenyl did not significantly decrease LPS-induced cytokine gene expression at 4 h. MAO enzyme activity generates hydrogen peroxide as an end product and hydrogen peroxide can regulate cytokine expression (Kim et al., 2011). However, stimulation of SiHa cells with hydrogen peroxide (10 and 100 µM) had no effect on IL-6 and IL-1β gene expression and cell viability (Supplemental Figure S2A–C).

### Dopamine Receptor Signaling Does Not Regulate Cytokine Expression in SiHa Cells

**FIGURE 4:**
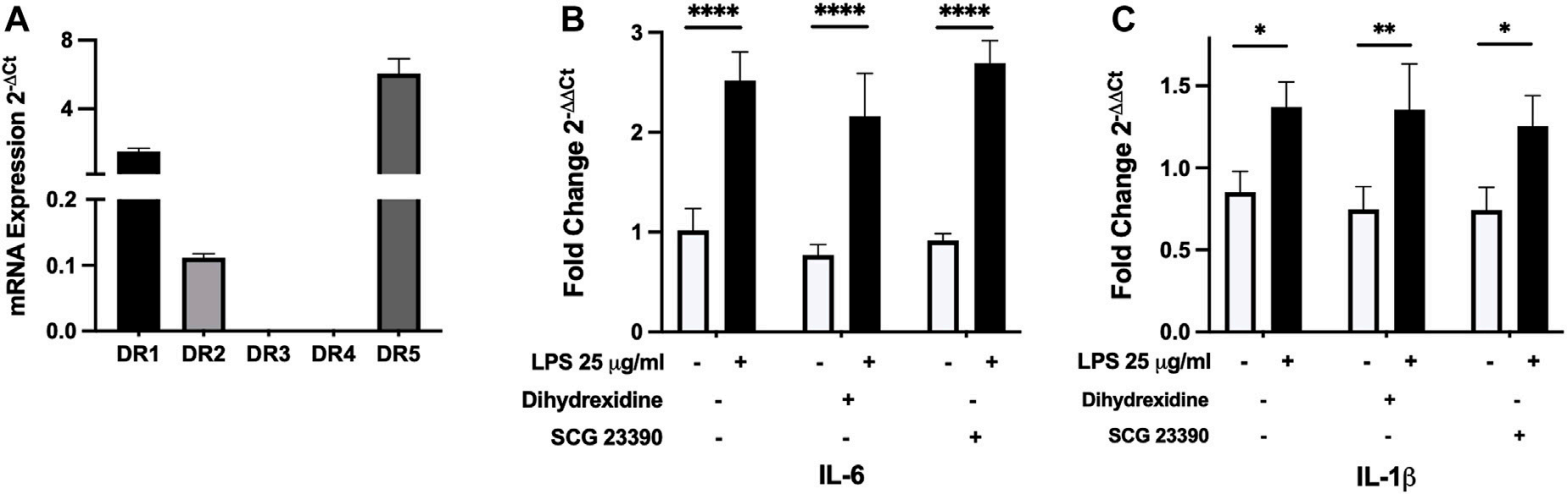
SiHa cells primarily express D1-like dopamine receptors but receptor-specific agonists/antagonists do not modulate LPS-induced IL-6 and IL-1β gene expression. **(A)** Using qRT-PCR, SiHa cells were found to express D1-like (DR1 and DR5) receptors and lower levels of the D2-like receptor (DR2). IL-6 **(B)** and IL-1β **(C)** gene expression in control (white bars) or LPS-treated cultures (black bars) were not altered by 1 µM dihydrexidine HCl (D1-like receptor agonist) or 1 µM SCG 23390 (D1-like receptor antagonists). The analysis was done using qRT-PCR. Data presented as mean ± SD (receptor expression, *n* = 3; agonist/antagonist, *n* = 4). *p < 0.05, **p < 0.01 and ****p < 0.0001.

MAO inhibitors, by their mechanism of action, can increase the availability of dopamine and this may signal through a variety of receptors. We first examined gene expression of dopamine DR1 to DR5 receptors in SiHa cells. D1-like receptors (DR1 and DR5) were more highly expressed than D2-like receptors. Of the D2-like receptor family, only DR2 expression was found (Figure 4A). We utilized targeted DR agonist/antagonists to examine whether altered receptor signaling could regulate LPS-induced cytokine expression. Interleukin-6 and IL-1β gene expression in control and LPS-treated cultures were examined using a D1-like agonist (dihydrexidine hydrochloride) and D1-like antagonist (SCG 23390 hydrochloride) (Kuzhikandathil and Oxford, 2002; Marino and Levy, 2019). LPS-induced IL-6 and IL-1β gene expression but the D1-like receptor agonist and antagonist had no effect on their expression (Figures 4B,C). As low DR2 expression was also found, we stimulated cultures with either a D2 agonist (sumanirole maleate) or antagonist (L-741,626) (Pillai et al., 1998; Marino and Levy, 2019). Neither affected control or LPS-induced cytokine expression (Supplemental Figure S3). Subsequently, we examined control ± LPS-treated cell cultures to determine whether SiHa cells synthesize dopamine. Dopamine is synthesized in a multi-step process from tyrosine to L-DOPA by tyrosine hydroxylase and subsequent conversion of L-DOPA to dopamine by dopamine decarboxylase. We found no significant gene expression of either dopamine synthesis enzymes (data not shown).

### MAO-B Inhibitors Decrease cAMP-PKA/EPAC-Mediated IL-6 and IL-1β Gene Expression

**FIGURE 5:**
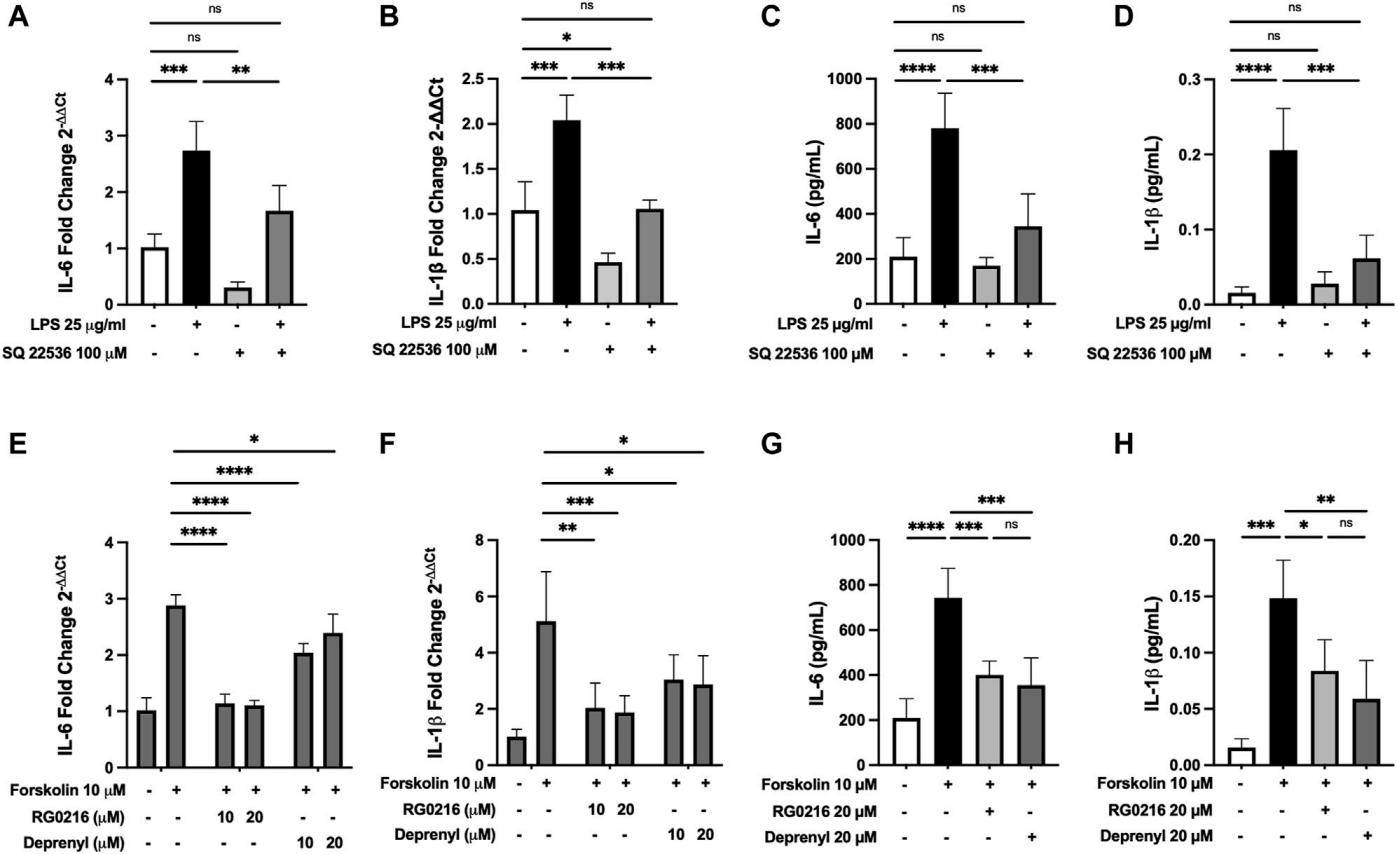
RG0216 and deprenyl downregulate cAMP-mediated induction of IL-6 and IL-1β gene and protein expression. Co-incubation of ± LPS-treated SiHa cell cultures with 100 µM SQ 22536 (an adenylyl cyclase inhibitor) effectively decreased LPS-induced pro-inflammatory gene expression for IL-6 **(A)** and IL-1β **(B)**. The analysis was done using qRT-PCR (*n* = 4). SQ 22536 decreased LPS-induced IL-6 **(C)** and IL-1β **(D)** protein expression as well (*n* = 5). Conversely, 10 µM forskolin (an adenylyl cyclase activator) alone significantly induced gene expression of IL-6 **(E)** and IL-1β **(F)** and these forskolin-induced pro-inflammatory cytokines were significantly reduced by both RG0216 and deprenyl. The analysis was done using qRT-PCR (*n* = 5). Forskolin-induced IL-6 **(G)** and IL-1β **(H)** protein expression was also reduced by RG0216 and deprenyl (*n* = 5). Data presented as mean ± SD. ns, not significant, *p < 0.05, **p < 0.01, ***p < 0.001 and ****p < 0.0001.

As cAMP is a potent mediator of immune responses and LPS can increase cAMP activation, we hypothesized that RG0216 and deprenyl may modulate cAMP signaling. We first examined cAMP involvement in LPS-induced IL-6 and IL-1β expression using a cAMP inhibitor (SQ 22536) (Weber et al., 2018). This cAMP inhibitor had no effect on control IL-6 expression but reduced control IL-1β gene expression. However, targeted inhibition of cAMP activation reduced LPS-induced IL-6 and IL-1β gene expression to respective controls (Figures 5A,B). LPS induction of IL-6 and IL-1β protein expression was also reduced by inhibition of cAMP activation (Figures 5C,D). These data support that, in SiHa cells, IL-6 and IL-1β upregulation by LPS is mediated by cAMP activation. We next examined the effects of RG0216 and deprenyl on cytokine expression induced by forskolin, a targeted cAMP activator (Kim et al., 2005). Interleukin-6 and IL-1β gene expression were highly induced by forskolin, and RG0216 and deprenyl both significantly reduced cAMP-mediated IL-6 and IL-1β gene expression (Figures 5E,F). In concert, forskolin induction of IL-6 and IL-1β protein expression was also reduced by RG0216 and deprenyl (Figures 5G,H). These data support that these MAO-B selective inhibitors have an inhibitory effect along the cAMP signaling pathway.

**FIGURE 6:**
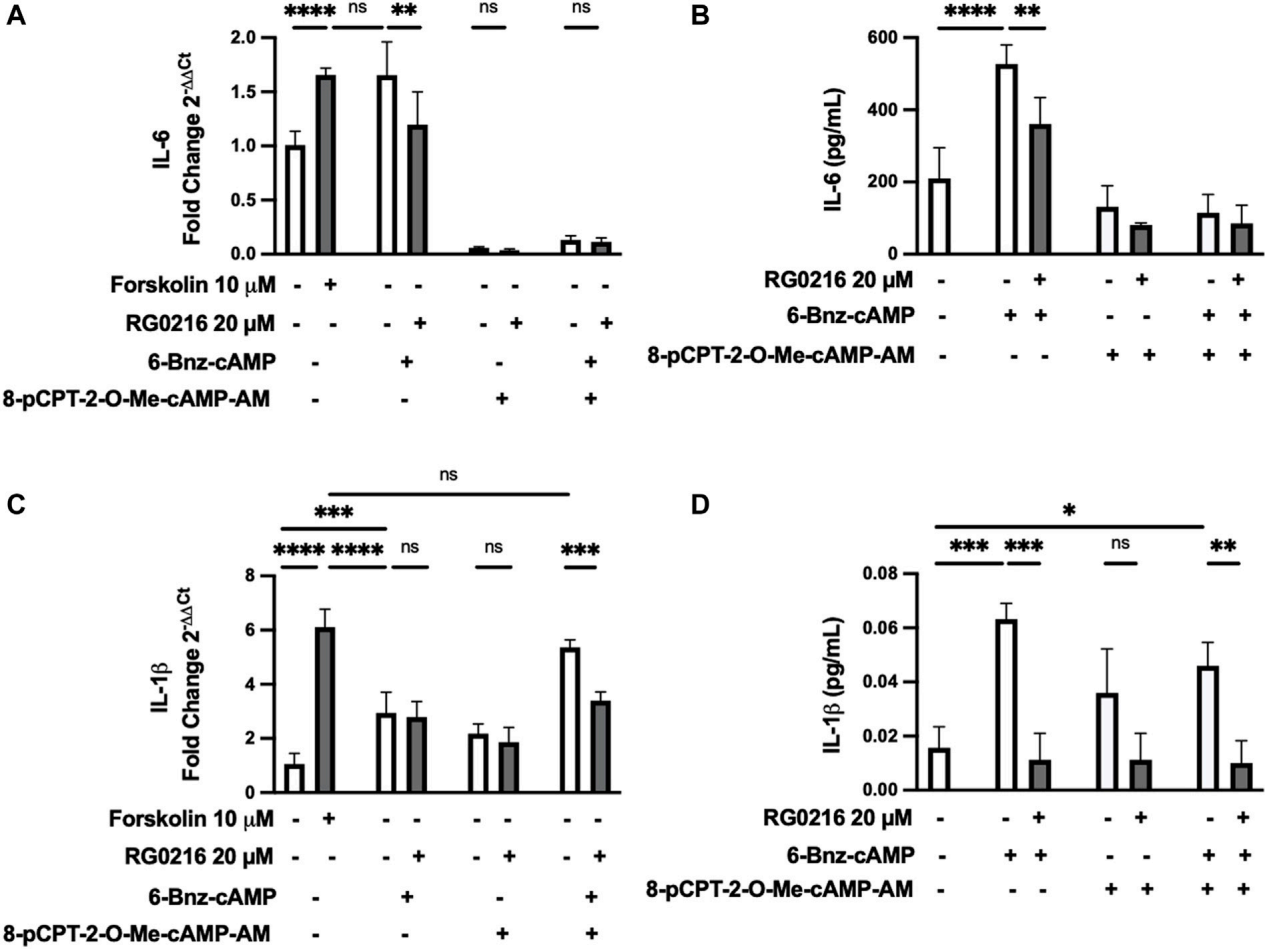
IL-6 and IL-1β gene and protein expressions are uniquely upregulated by PKA and EPAC activation but both are decreased by RG0216. **(A)** IL-6 gene expression was significantly induced by PKA activation (100 µM 6-BNZ-cAMP). EPAC selective activation (10 µM 8-pCPT-2-O-Me-cAMP-AM) fully inhibits IL-6 expression in controls and negates PKA-induced IL-6 expression. RG0216 decreased PKA-induced IL-6 gene expression. **(B)** RG0216 also decreased PKA activation-induced effect on IL-6 protein expression. **(C)** IL-1β gene expression was partially induced with PKA selective activation but not by EPAC selective activation. Co-stimulation with PKA and EPAC activator induced IL-1β gene expression and this increase was reduced with RG0216. Forskolin was the positive control. **(D)** RG0216 reduced PKA/EPAC- and PKA alone-mediated effects on IL-1β protein expression. EPAC-activation alone had no effect on IL-1β protein expression. Data presented as mean ± SD (qRT-PCR, *n* = 5, protein, ≥ 3). ns, not significant, *p < 0.05, **p < 0.01, ***p < 0.001 and ****p < 0.0001.

PKA and EPAC are critical downstream mediators of cAMP signaling and each can have diverse, individual and interactive signaling effects. We examined the induction of IL-6 and IL-1β gene and protein expression by PKA and EPAC selective activators and the effect of RG0216 on this activation. Cultures were treated with a PKA selective activator (6-Bnz-cAMP) and/or EPAC selective activator (8-pCPT-2-O-Me-cAMP-AM) and compared to forskolin-treated cultures (positive control). Targeted pathway activation uniquely regulated IL-6 and IL-1β (Figure 6). Selective PKA activation fully induced IL-6 gene expression to the level induced by forskolin (positive control), while EPAC selective activation exerted a dominant negative regulatory effect when used alone and fully inhibited PKA-induced IL-6 expression such that it was reduced below control (Figure 6A). PKA and EPAC activation showed similar effects for IL-6 protein expression (Figure 6B). In both gene and protein studies, RG0216 significantly reduced PKA activation-induced IL-6 expression. In contrast, PKA selective activation partially induced IL-1β gene expression but this was below the expression induced by forskolin (positive control). EPAC activation alone had no effect on the upregulation of IL-1β gene expression. However, when PKA and EPAC activators were used together, IL-1β expression was induced to the same level as the forskolin positive control. RG0216 reduced PKA/EPAC-induced IL-1β gene expression (Figure 6C). PKA alone and PKA/EPAC-induced activation also increased IL-1β protein expression at 12 h and RG0216 effectively reduced this expression (Figure 6D).

## Discussion

Prescriptive use of MAO inhibitors and their association with reducing inflammation has been described. Historically, two clinical reports described small populations of patients taking MAO inhibitors for depression and reporting clinical improvement in their Crohn’s disease and reduced arthritic joint pain and stiffness (Lieb, 1983; Kast, 1998). MAO-A, MAO-B and MAO-A/B inhibitors have all been shown to reduce cytokine expression in CNS-associated disease models (Bielecka et al., 2010; Fernandez et al., 2011; Panarsky et al., 2012; Trudler et al., 2014; Liu et al., 2020; Tomaz et al., 2020) and non-CNS-associated disease models that examined either whole blood, bone stromal cells, periodontal tissues, lung tissues or bone marrow-derived macrophages samples (Lin et al., 2000; Ekuni et al., 2009; Chaaya et al., 2011; Cui et al., 2017; Wu et al., 2017; Cui et al., 2020; Sanchez-Rodriguez et al., 2021). In general, MAO-A, -B and -A/B inhibitors have been shown to reduce TNF-α, IL-1β, IL-8, IL-6, monocyte chemoattractant protein-1, cytokine-induced neutrophil attractant-1 expression and increased expression of the anti-inflammatory cytokine IL-10 (Ostadkarampour and Putnins, 2021).

RG0216 is a reversible MAO-B selective inhibitor with reduced transport across the blood–brain barrier (Gealageas et al., 2018). We tested its anti-inflammatory potential using LPS-treated cervical mucosal cells (SiHa) that only express MAO-B protein (Figure 1). LPS is often used as an inducer of immune responses in MAO inhibitor studies examining cytokine regulation (Lin et al., 2000; Ekuni et al., 2009; Sanchez-Rodriguez et al., 2021). LPS-induction of IL-8, IL-6, IL-1β and TNF-α expression in SiHa cells was consistent with previous studies (He et al., 2016; He et al., 2017). However, RG0216 consistently decreased LPS-upregulated IL-6 and IL-1β gene and protein expression, suggesting that a common signaling point may have been inhibited. Understanding the mechanism by which this is mediated is important.

MAO inhibitors, by their mechanism of action, decrease metabolic end products or increase biogenic amines and both outcomes may impact cell signaling (Ostadkarampour and Putnins, 2021). Hydrogen peroxide is a significant metabolic end product that is also an oxidative stress molecule involved in inflammation-associated cell signaling. However, excessive accumulation of hydrogen peroxide is associated with chronic inflammatory diseases (Wittmann et al., 2012; Pravda, 2020). Lipopolysaccharide induction of MAO-A and -B expression has been demonstrated in rat aorta organ cultures and was associated with increased hydrogen peroxide, which was reduced with MAO inhibition (Ratiu et al., 2018). Gastric epithelial cell cultures treated with hydrogen peroxide showed increased IL-8 expression (Kim et al., 2011). However, in this study, LPS did not induce MAO-B expression in SiHa cells (Figure 1) and direct stimulation of cells with hydrogen peroxide (Supplemental Figure S2) had no effect on IL-6 and IL-1β expression. RG0216-mediated reduction in cytokine expression did not appear to be reflective of drug-induced change in hydrogen peroxide generation.

MAO inhibitors also increase biogenic amines such as dopamine and this increase can modify immune responses (Ostadkarampour and Putnins, 2021). Dopamine signaling can act in a receptor-dependent and also receptor-independent manner. Dopamine receptors are grouped into two subclasses: D1-like (DR1, DR5) and D2-like (DR2, DR3, DR4), are expressed in CNS and non-CNS locations and their expression varies based on tissue type (Beaulieu and Gainetdinov, 2011; Handa et al., 2019). Epithelial cells do express DR1, DR2 and DR3 (Mao et al., 2015; Matsuyama et al., 2018; Reyes-Resina et al., 2020). SiHa cells were found to express D1-like (DR1, DR5) receptors at higher levels than the DR2. Dopamine receptor-mediated cell signaling has been extensively examined using selective dopamine receptor agonists and antagonists (Heidegger, 1987; Yang et al., 2016; Handa et al., 2019). However, D1-like and DR2 specific agonists and antagonists had no regulatory effect on IL-6 and IL-1β cytokine expression in control and LPS-treated SiHa cell cultures. Dopamine-mediated receptor independent signaling has also been described. In this model, dopamine attenuated LPS-induced cytokine expression by the formation of dopamine quinone. Dopamine quinone forms by its auto-oxidation and subsequent covalent conjugation to cysteine residues on proteins. LPS-induced NF-κB translocation and cytokine expression were reduced by dopamine quinone (Yoshioka et al., 2020). Cells of the peripheral and central nervous system express dopamine; however, a number of immune cells synthesize dopamine as well (Pinoli et al., 2017). Alveolar epithelial cells also synthesize dopamine but require the addition of L-DOPA (Adir et al., 2004). Dopamine is synthesized in a multi-step process from tyrosine to L-DOPA by tyrosine hydroxylase and subsequent conversion of L-DOPA to dopamine by dopamine decarboxylase (Eisenhofer et al., 2004; Pinoli et al., 2017). We found no significant expression of these dopamine synthesis enzymes. Taken together, RG0216-mediated regulation of LPS-induced cytokine expression was not reflective of altered dopamine-mediated receptor-dependent nor -independent signaling.

Cyclic AMP is a second messenger that regulates a multitude of cellular processes and is increased by LPS (Moon et al., 2011a; Moon et al., 2011b). We found LPS-induction of IL-6 and IL-1β gene and protein expression was fully inhibited and returned to control levels by co-treatment with a selective inhibitor of cAMP (SQ 22536) (Figures 5A–D). Therefore, in SiHa cells, LPS-induction of IL-6 and IL-1β is fully cAMP dependent. Forskolin (cAMP activator) significantly induced IL-6 and IL-1β gene and protein expression and these data are consistent with previous studies which showed that forskolin induced IL-6 expression (Grassl et al., 1999; Wang et al., 2010; Nilsson et al., 2017). RG0216 was highly effective at reducing forskolin-induced cytokine gene expression; however, by 12 h, cytokine protein expression was equally reduced by both RG0216 and deprenyl (Figure 5E–H).

PKA and EPAC are both well recognized downstream effectors of cAMP activation. PKA and EPAC can both activate/inhibit the Ras/Rap-Raf-MEK-ERK pathway with their subsequent independent, synergic or antagonistic interactions that ultimately regulate and modulate cell signaling (de Rooij et al., 1998; Stork and Schmitt, 2002; Bos, 2003, Bos, 2006; Cheng et al., 2008; Miningou and Blackwell, 2020). PKA can either activate or inhibit ERK and EPAC activates ERK through Rap1 (Stork and Schmitt, 2002; Bos, 2003; Miningou and Blackwell, 2020). PKA and EPAC were both involved in the regulation of IL-6 and IL-1β expression in SiHa cells but did so in uniquely different ways. Specifically, PKA activation alone induced IL-6 expression and EPAC activation alone had a dominant negative regulatory role on IL-6 and fully negated PKA-induced gene and protein expression. This is consistent with a previous study which found that EPAC activation inhibited TH_2_ cytokine expression and airway hyperresponsiveness in an acute asthma mouse model (Chen et al., 2019). In sharp contrast, PKA alone marginally induced IL-1β gene expression and EPAC alone had little effect but together fully induced IL-1β expression. This is consistent with previous work which showed that ERK activation required EPAC and PKA-mediated signaling (Vossler et al., 1997; Bos, 2003). Changes in IL-1β protein expression by PKA- and EPAC-mediated signaling paralleled gene expression studies to a large degree. RG0216 reduced PKA/EPAC-induced IL-1β protein expression. However, PKA stimulation alone also induced IL-1β protein expression by 12 h and this was also reduced with RG0216. Possibly EPAC co-stimulation enhanced PKA-induced IL-1β gene transcription, but PKA alone was able to induce IL-1β expression with extended culture times. Collectively, LPS-induced IL-6 and IL-1β cytokine expression was mediated through cAMP-PKA/EPAC signaling and RG0216 significantly reduced both. The finding that both cytokines are significantly decreased by RG0216 while each was uniquely regulated by PKA and EPAC activation supports a hypothesis that the RG0216 point of inhibitory action is not at the level of cAMP but lies downstream of PKA and EPAC.

A number of papers have examined MAO inhibitor-mediated cell signaling that is involved in cytokine expression. Tranylcypromine (MAO-A/B inhibitor) did not alter LPS-mediated NF-*κ*B signaling in the rat hippocampal region but prevented LPS-mediated reduction in cAMP response element binding protein (CREB) phosphorylation (Tomaz et al., 2020). Ladostigil (MAO-A/B inhibitor) reduced LPS-induced TNF-α, IL-6 and IL-1β in the rat parietal cortex and in glial cell culture, and also reduced LPS-induced p38 and ERK1/2 mitogen-activated protein kinase activation and NF-κB translocation to the nucleus (Panarsky et al., 2012). Moclobemide (MAO-A inhibitor) reduced LPS-induced IL-1β and TNF-α in glial cell cultures and reduced NF-κB translocation to the nucleus (Bielecka et al., 2010). In addition, smoke-induced upregulation of pro-inflammatory cytokines in cell culture and bronchoalveolar lavage were reduced with deprenyl (MAO-B inhibitor) and deprenyl reduced p38 and ERK 1/2 phosphorylation and NF-κB translocation into the nucleus (Cui et al., 2017; Cui et al., 2020). The described MAO inhibitor reduction in cytokine expression was often associated with altered ERK activation and CREB, NF-*κ*B transcription factor signaling. PKA/EPAC signaling lies upstream of these regulators of cytokine expression. In LPS-stimulated microglial cell cultures, the expression of IL-6 and TNF-α were regulated by cAMP/PKA/CREB, EPAC regulates NF-κB activation in macrophages and both EPAC and PKA can regulate ERK activation (Mayr and Montminy, 2001; Moon et al., 2011a; Wang B. et al., 2018; Miningou and Blackwell, 2020). RG0216 inhibition of PKA/EPAC signaling may potentially decrease transcription factor activation as has often been described for existing MAO inhibitors. Deprenyl inhibition of cAMP-induced cytokine expression does provide some support for this hypothesis.

RG0216 is a reversible and selective MAO-B inhibitor, developed with reduced *in vivo* transport into the brain. It is a less potent inhibitor of MAO-B than deprenyl but was less rapidly metabolized in plasma, liver microsomes and hepatocytes (Gealageas et al., 2018). Of interest was the finding that even though RG0216 was less potent at inhibiting MAO-B activity when compared to deprenyl, it was more efficacious at reducing LPS-induced cytokine gene expression (Figure 3A) as well as forskolin-induced cytokine gene expression (Figures 5E,F) at equimolar concentrations; however, by 12 h, cytokine protein expression (Figures 5G,H) was equally reduced by both MAO-B inhibitors. Possibly the differences found between gene and protein data for both MAO-B inhibitors is reflective of their different rates of metabolism or possibly reflect that RG0216 and deprenyl are either reversible or irreversible inhibitors, repectively. These differences may have had a differential effect on transcription. In summary, RG0216 effectively reduced LPS-induced expression of IL-6 and IL-1β. Cytokine reduction was not reflective of hydrogen peroxide nor dopamine-mediated signaling but we present a novel finding that the inhibitory action of RG0216 was a reflection of drug-mediated inhibition of cAMP-PKA/EPAC signaling. This is the first report demonstrating MAO-B inhibitor downregulation of pro-inflammatory cytokine expression is effective through mediating cAMP signaling. Further studies are planned to examine the anti-inflammatory therapeutic efficacy of RG0216 using an *in vivo* LPS-mediated inflammatory disease model, and to further investigate the mechanism by which RG0216 inhibits PKA/EPAC-mediated signaling and how this differs from existing MAO inhibitors.

## Data Availability

The original contributions presented in the study are included in the article/Supplementary Material, further inquiries can be directed to the corresponding author.
