# Supplementary figures and images for: Monoamine Oxidase-B Inhibitor Reduction in Pro-Inflammatory Cytokines Mediated by Inhibition of cAMP-PKA/EPAC Signaling
Source: Front Pharmacol. 2021 Nov 17;12:741460. doi: 10.3389/fphar.2021.741460 (PMC8635787; doi:10.3389/fphar.2021.741460)

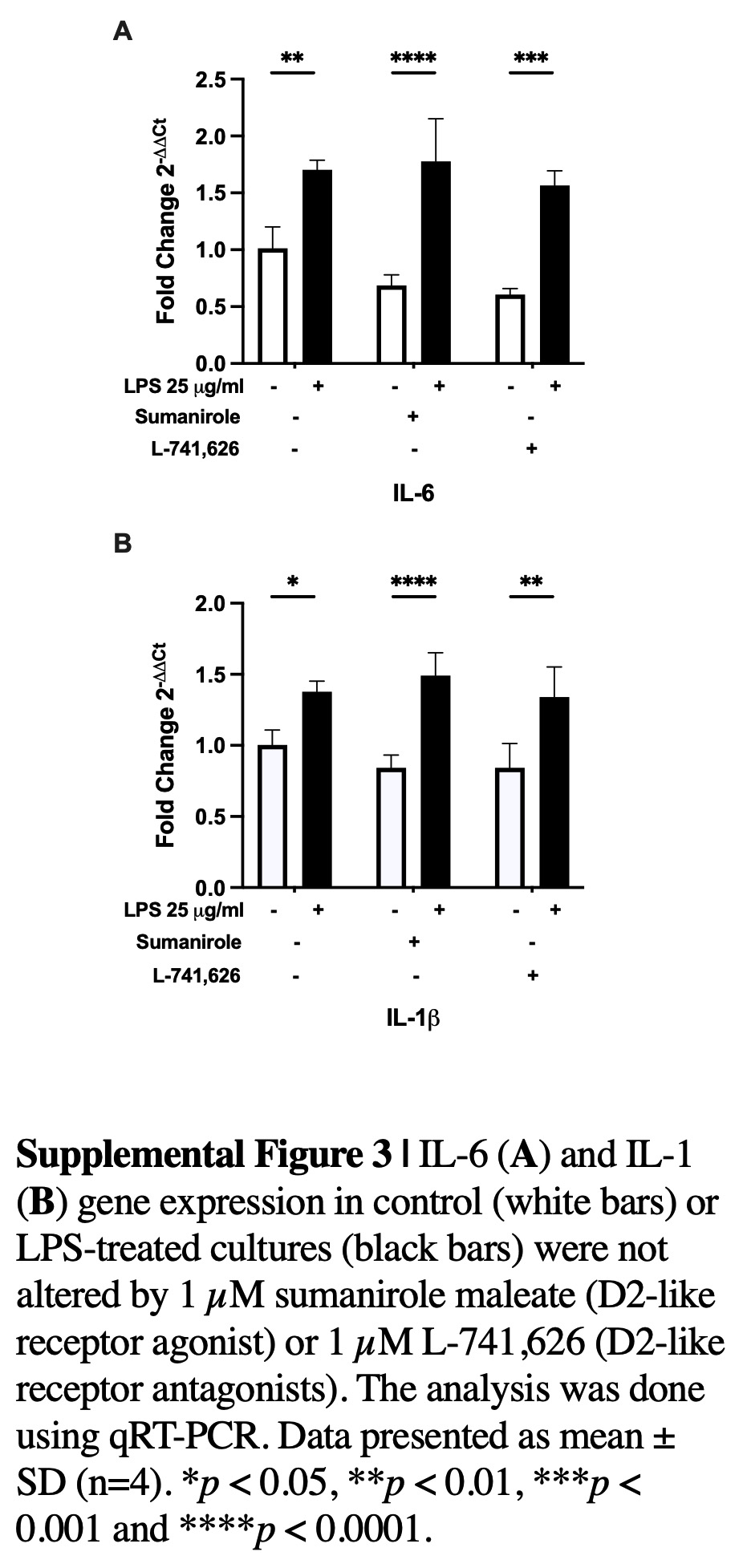

Supplement: Supplementary file 1 [file Image3.jpg]

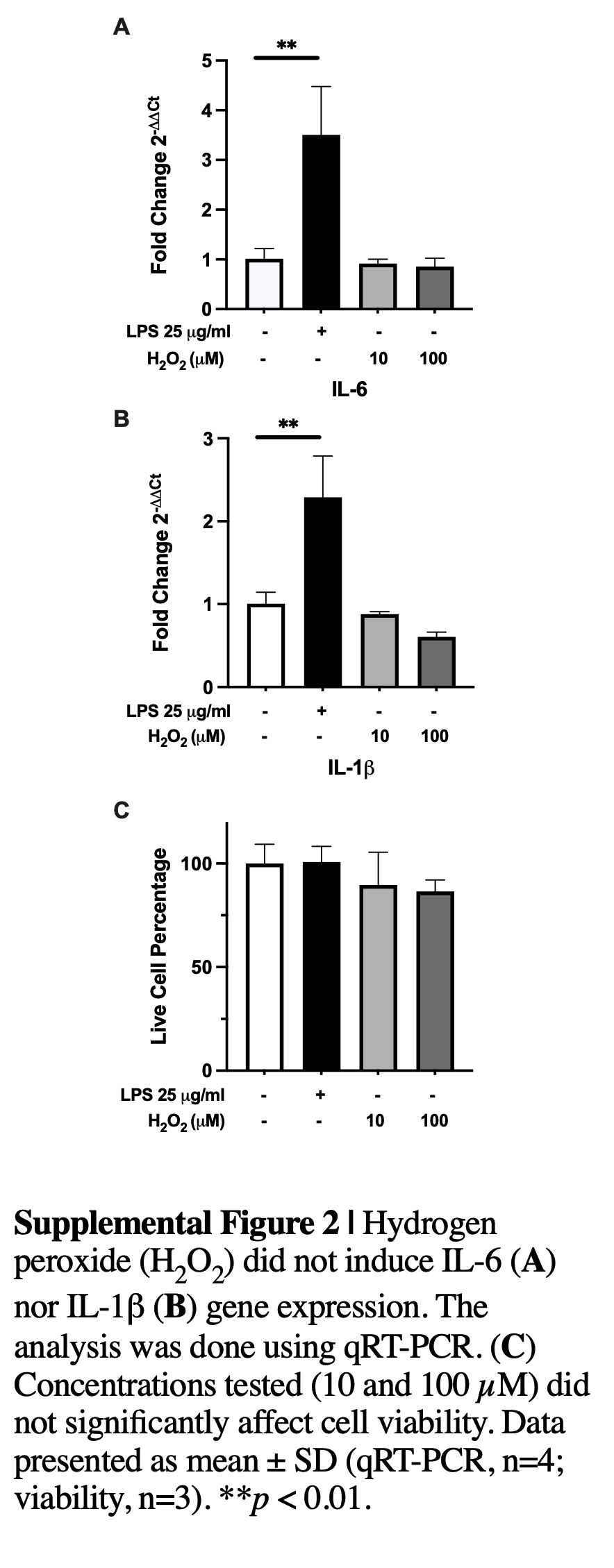

Supplement: Supplementary file 2 [file Image2.jpg]

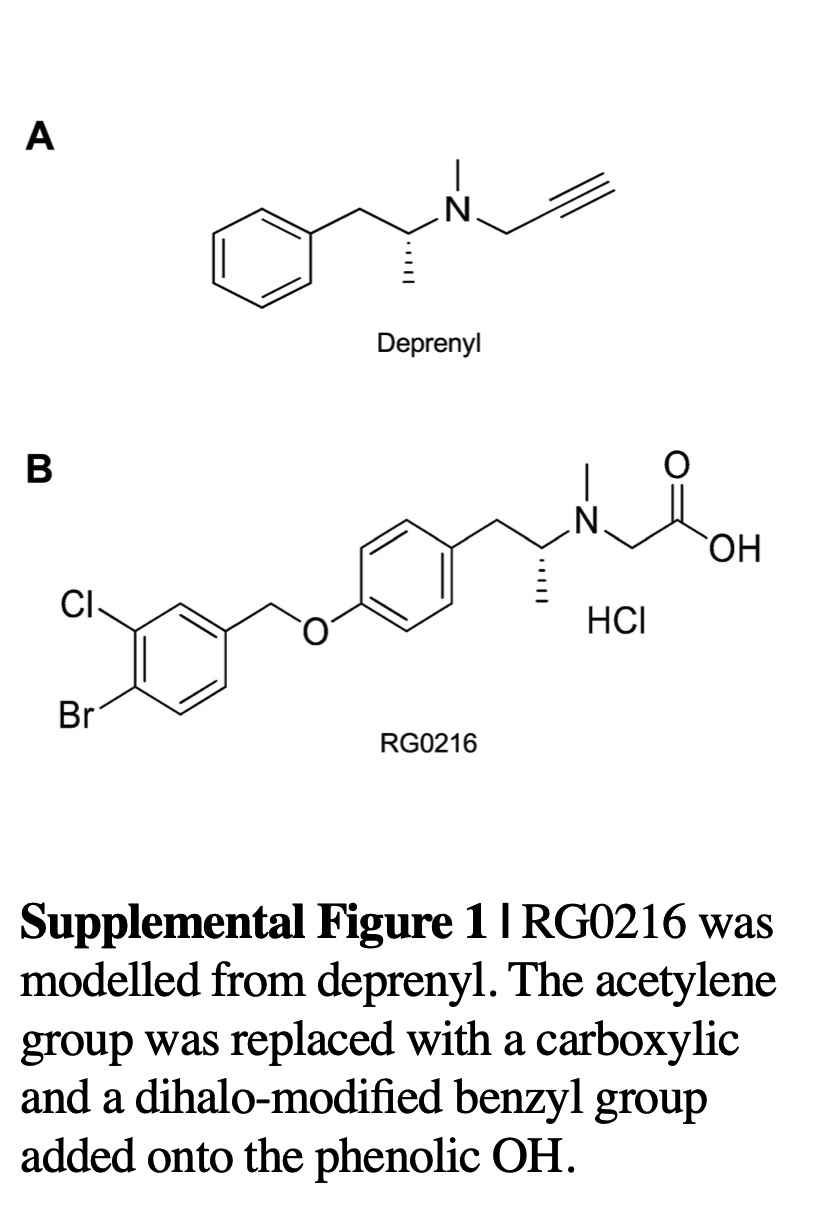

Supplement: Supplementary file 3 [file Image1.jpg]
